# A Review of Recent Deep Learning Approaches in Human-Centered Machine Learning

**DOI:** 10.3390/s21072514

**Published:** 2021-04-03

**Authors:** Tharindu Kaluarachchi, Andrew Reis, Suranga Nanayakkara

**Affiliations:** Augmented Human Lab, Auckland Bioengineering Institue, The University of Auckland, 70 Symonds Street, Grafton, Auckland 1010, New Zealand; andrew@ahlab.org (A.R.); suranga@ahlab.org (S.N.)

**Keywords:** human-centered machine learning, HCML, HCAI, human-centered artificial intelligence, Deep Learning

## Abstract

After Deep Learning (DL) regained popularity recently, the Artificial Intelligence (AI) or Machine Learning (ML) field is undergoing rapid growth concerning research and real-world application development. Deep Learning has generated complexities in algorithms, and researchers and users have raised concerns regarding the usability and adoptability of Deep Learning systems. These concerns, coupled with the increasing human-AI interactions, have created the emerging field that is Human-Centered Machine Learning (HCML). We present this review paper as an overview and analysis of existing work in HCML related to DL. Firstly, we collaborated with field domain experts to develop a working definition for HCML. Secondly, through a systematic literature review, we analyze and classify 162 publications that fall within HCML. Our classification is based on aspects including contribution type, application area, and focused human categories. Finally, we analyze the topology of the HCML landscape by identifying research gaps, highlighting conflicting interpretations, addressing current challenges, and presenting future HCML research opportunities.

## 1. Introduction

**Figure 1:**
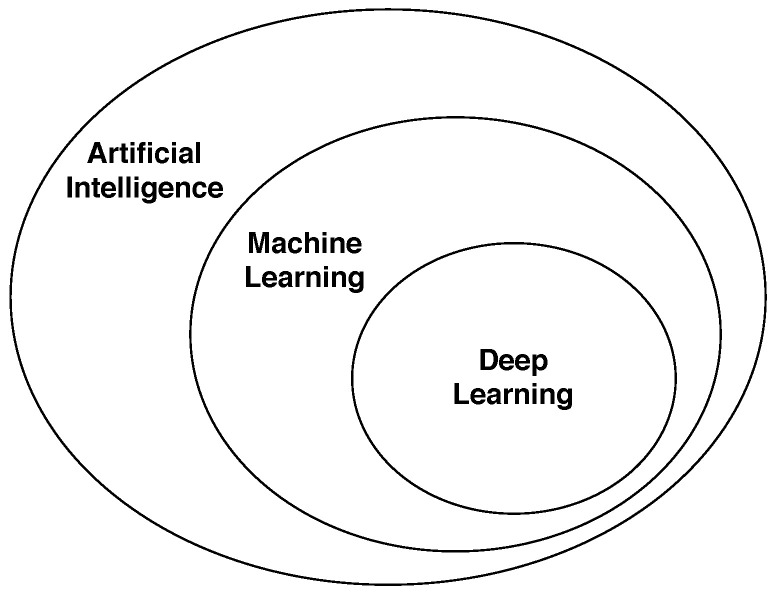
Overview of Artificial Intelligence. In modern-day practice, Deep Learning (DL) and Machine Learning (ML) are commonly referred to as Artificial Intelligence (AI), even though AI also includes rule-based simpler techniques. In this paper, the term AI refers to ML and DL.

Artificial Intelligence (AI) represents the broad spectrum of automated decision making from conditional logic to Neural Networks. Decisions or predictions made using data-driven techniques fall into Machine Learning (ML), a subset of AI. The subset of Machine Learning techniques that use Deep Neural Networks (DNN) is called Deep Learning (DL) (see Figure 1).

Over the last two decades, Artificial Intelligence (AI) research publications have grown to account for 9% of all conference publications and 3% of all journal publications [1]. The majority of this AI research typically explores developing algorithms and optimizing technologies with an emphasis on performant models benchmarked on accuracy. Apart from academic research, Machine Learning, Artificial Intelligence, and Deep Learning are incredibly pervasive within data-abundant industries. These same industries have built products and services atop an AI back-end with varying success. As the integration of performant models within the industry continues, there is an equally increasing requirement to analyze and improve the translation from the algorithmic model to end-user requirement.

### 1.1. Rise of Human-Centered Machine Learning (HCML)

**Figure 2:**
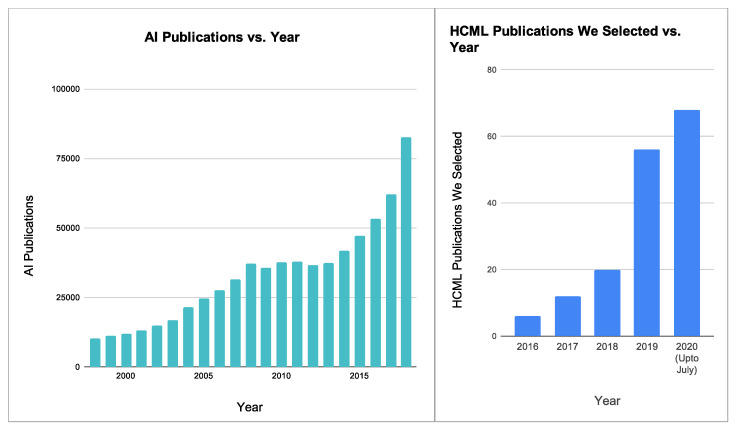
Rise of AI-related publications and Human-Centered Machine Learning (HCML) publications.

HCML terminology has been used in publications over a decade ago. In an assistive tool [2], Balasubramanian et al. claimed they introduced the term “Human-Centered Machine Learning Algorithms” through their investigation on human-in-the-loop ML systems. However, the HCML term started to gain and accelerate popularity in the mid-2010s after the modern Deep Learning era started. The average user does not yet understand the capabilities, limitations, and inner workings of AI. Therefore users have reported concerns [3,4] regarding explainability, user experience (UX) with the interfaces, user-data privacy, security, reliability, and dependability of AI systems. The need to solve these issues has evolved into the field of Human-Centered Machine Learning (HCML). Recognizing that algorithm optimization and innovative neural network architectures alone do not solve usability and adoptability problems, the HCML field seeks to elevate ML systems’ user-centered development. Reference to HCML terminology now exists in formal and informal forums, such as AI publications, workshops, conferences [5], and blog posts and articles from AI-focused corporations (see Figure 2).

HCML emerged as the research area investigating the methods of aligning machine learning systems with human goals, context, concerns, and ways of work [5]. Several different terminology and abbreviations have emerged alongside the many institutions researching the user experience aspect of artificial intelligence. The widespread use of ML and AI interchangeably has led institutions to use either of the terms Human-Centered Machine Learning (HCML) or Human-Centered Artificial Intelligence (HCAI or HAI). Several researchers have also started labeling this field the UX of AI (see Section 2 for detailed description). However, all these terms’ underlying principle remains the same, which is developing usable and adaptable, ‘Human-in-the-Loop’ machine learning systems.

Some of the most common non-expert-user issues are explainability [6,7,8,9,10], interpretability [11,12,13,14], privacy and security [15,16,17,18,19,20], reliability [21,22,23,24,25], and fairness [26,27,28,29,30,31]. These categories emerged as contributing research areas of HCML, and each plays a role in the broader goal of improving the usability and adoptability of AI systems. It is important to note that these research areas are not new but instead are aligned with the underlying motivations of HCML. The growing demand to adapt ML systems to real-life use cases requires timely and in-depth HCML research; however, it is imperative to survey the landscape of previous works in this emerging field.

### 1.2. Scope and Contribution

**Figure 3:**
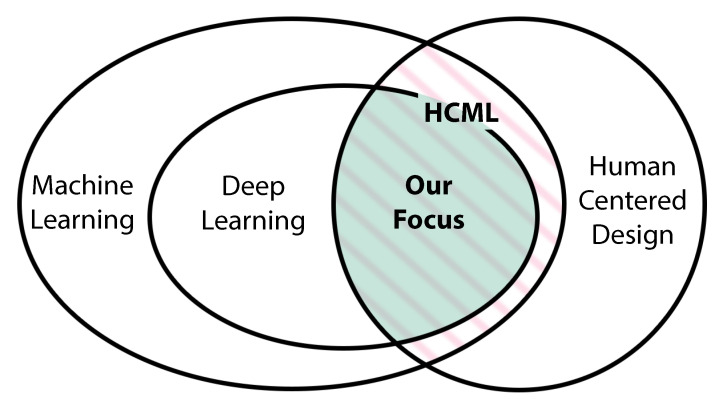
Human-Centered Machine Learning (HCML) is the intersection of Machine Learning and Human-Centered Design (marked with pink stripes). In this paper, our focus lies in HCML approaches related to Deep Learning as marked by the green area.

This paper analyzes HCML work related to Deep Learning (as highlighted in Figure 3). HCML emerged in the mid-2010s with increasing popularity, mainly due to the widespread adaptation of Deep Learning. The rapid development of hardware to power Deep Learning algorithms enabled novel neural network architecture research. Deep Learning algorithms’ complex nature was the main reason for the rise of some sub-topics in HCML, such as explainability and interpretability. The exponential growth of Deep Learning and the existence of sub research areas within HCML generated diverse research from algorithm development and optimization to user studies research. The research focus varies, focusing on the algorithm, user-studies, concepts, or a mixture of these.

There has been no prior work done to investigate and detail Deep Learning related prior work within the HCML domain to the best of our knowledge. Therefore, in this work, we analyze recent approaches in HCML related to Deep Learning. In summary, our key contributions of this work are:

- Working definition of HCML: Many leading research groups have interpreted HCML through the lens of their respective institutions. Those interpretations present multiple perspectives on the totality of HCML. Therefore, we have analyzed these interpretations and subsequently derived a working definition (see Section 2) that envelopes the prevailing work in the HCML field. We validated the final definition through consultation with leading academics and industry experts who are spearheading HCML research.
- Systematic Literature Review: We employ a Systematic Literature Review (SLR) technique to filter and select publications, as explained in Section 3. We categorize and critically analyze 162 publications based on four criteria. Prior work stems from a range of domains such as medicine and software engineering, and the nature of these publications takes several forms. Some focus on specific user-concerns while others highlight general aspects within HCML. These variations paved the way for us to build a taxonomy of HCML publications (see Section 4).
- Challenges and opportunities for HCML research: Given the emerging nature of the HCML field, research conducted in HCML faces challenges from several directions. Our analysis across the defined scope of HCML reveals opportunities for future research. We discuss these points in detail in the discussion section (see Section 5).

## 2. Defining HCML

Human-Centered Machine Learning, also referred to as Human-Centered Artificial Intelligence (HCAI or HAI), is gaining popularity due to the concerns raised by influential technology firms and research labs about the human context. A workshop in conjunction with the Conference on Human Factors in Computing Systems in 2016 [5] explained that HCML should explicitly recognize the human aspect when developing ML models, re-frame machine learning workflows based on situated human working practices and explore the co-adaptation of humans and systems. In early 2018, Google Design (https://design.google/library/ux-ai/, accessed on 1 April 2021) published an article noting that HCML is the User Experience (UX) of AI. Referring to a real consumer ML product, Google highlighted how ML could focus on human needs while solving them in unique ways that are only possible through ML. Several research projects (https://hcai.mit.edu/, accessed on 1 April 2021) by the Massachusetts Institute of Technology (MIT) on self-driving technologies called their approach Human-Centered Artificial Intelligence. The MIT team recognized both the development of AI systems that are continuously learning from humans and the parallel creation and fulfillment of a human-robot interaction experience. In 2019, the Stanford Institute for Human-Centered Artificial Intelligence (https://hai.stanford.edu/, accessed on 1 April 2021) was initiated with the goal of improving AI research, education, policy, and practice. They recognized the significance of developing AI technologies and applications that are collaborative, augmentative, and enhance human productivity and quality of life. A workshop (https://sites.google.com/view/hcml-2019, accessed on 1 April 2021) held in 2019 with the Conference on Neural Information Processing Systems for Human-Centered Machine Learning focused on the interpretability, fairness, privacy, security, transparency, accountability, and multi-disciplinary approach of AI technologies. Started in 2017, Google People + AI Research initiative (https://pair.withgoogle.com/, accessed on 1 April 2021) published a 2019 book presenting guidelines for building human-centered ML systems. This team is researching the full spectrum of human interactions with machine intelligence to build better AI systems with people.

Considering the scope of the HCML/HCAI prior work and publications by leading industry and academic institutions, we derived a definition for HCML that covers the breadth of this existing work. We validated the definition using feedback from several researchers working in the same domain and further validated with some influential researchers in leading academia and industrial institutions’ Human-Centered AI research teams.

***Human-centered Machine Learning (HCML): Developing adaptable and usable Machine Learning systems for human needs while keeping the human/user at the center of the entire product/service development cycle.***

- *“Adaptable” includes adding features such as explainability, interpretability, fairness, privacy, security, transparency, and accountability.* 
- *“Usable” refers to the UX of AI, including system usability and user burden.* 
- *“Human needs” implies the significance of the problems we are selecting to solve with AI.* 
- *“Entire development cycle” includes all steps from conceptualization to maintenance, which extends from Human-Centered Design to working systems that are continuously learning.* 

There is a natural incentive to research all the principles mentioned previously; however, this is seldom achieved in practice. In individual research, the entire development life-cycle is only partially detailed, possibly due to emphasis on the focused technicalities of the research. Therefore, we selected research that demonstrated one or more design elements matching the above definition of HCML research.

**Figure 4:**
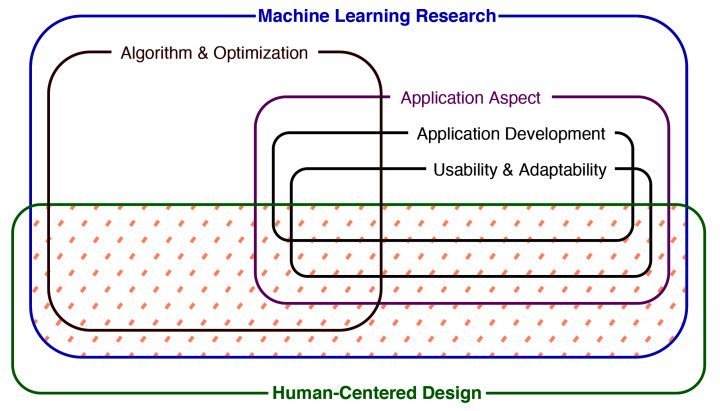
Human-Centered Machine Learning (marked with dashed lines) research lays over a broad spectrum, as shown here. The intersection of Machine Learning Research and Human-Centered Design is the domain we identify as Human-Centered Machine Learning.

As shown in Figure 4, HCML work lies across many aspects of Machine Learning. We define algorithmic work related to HCML as Back-End HCML and work with interactions with humans as Front-End HCML. We excluded algorithm-centric back-end HCML papers as it would divert our focus away from the baseline HCML concepts. For instance, analyzing and classifying explainability algorithms is beyond this paper’s scope and may be reviewed in separate works, such as [32,33]. However, algorithmic contributions with Front-End HCML practices, such as user evaluations, were included.

## 3. Systematic Literature Review Approach

Systematic Literature Review (SLR) is a technique used to provide an extensive overview of the literature extracted by a repeatable and objective process. Although SLR was initially used in research within the domain of medicine, it is also gaining popularity in computer science [34,35,36,37]. We chose to follow the SLR approach when selecting literature on Human-Centered Machine Learning because it reduces the subjective nature of the process while widening the search area. Following the development and finalization of the working HCML definition, we conducted a three-phase SLR approach.

Phase 1:

We performed a domain-specific keyword and title search for “Human-Centered Machine Learning” and “Human-Centered Artificial Intelligence”. We employed Google Scholar (https://scholar.google.com/, accessed on 1 April 2021), ACM Digital Library (https://dl.acm.org/, accessed on 1 April 2021), and IEEE Xplore (https://ieeexplore.ieee.org/Xplore/home.jsp/, accessed on 1 April 2021) to sample a diverse collection of research. We downloaded 200 papers as the initial step. We went through the titles, keywords, and abstracts of all papers and selected 85 publications that matched.

Phase 2:

We used a specific selection process based on a set of specific inclusion and exclusion criteria to select papers. The selection process was to use five independent researchers working in the field of AI and HCML to vote for publications based on the inclusion and exclusion criteria, which is as follows:

- Included—Deep Learning papers published from 2016 (when HCML re-emerged)-May 2020.
- Included—Traits that matched our HCML definition of usability and adoptability.
- Included—Publications surveys and guidelines that are tagged as Human-Centered Machine Learning.
- Excluded—Work that did not contain real human/user inputs in one or more stages of development.

Equally weighted vote distributions from the researchers were used to select papers. From the initial 85 papers of the initial pool, this selection process picked 31 papers. These 31 papers were used as the basis for expanding the initial search base. We used additional HCML keywords, extracted from the selected papers, including “Human-AI interaction”, “user experience”, “user-centered design”, “empirical-study”, “end-user machine learning” and “machine learning”. Using these targeted keywords, we downloaded an additional 50 publications. After analyzing them, we identified a broad and diverse range of keywords used in HCML related work.

Phase 3:

In the third stage, in addition to the keyword search using search engines that we used for the first stage, we expanded our search to proceedings that typically have papers with Human-Centered Design and Machine Learning Research. This includes, CHI proceedings (https://sigchi.org/conferences/conference-history/chi/, accessed on 1 April 2021), UIST Proceedings (https://sigchi.org/conferences/conference-history/uist/, accessed on 1 April 2021), IUI proceedings (https://sigchi.org/conferences/conference-history/iui/, accessed on 1 April 2021), Neural Information Processing Systems proceedings (https://nips.cc/, accessed on 1 April 2021) and AAAI proceedings (https://www.aaai.org/, accessed on 1 April 2021). Since our focus is on Deep Learning and HCML, we downloaded all the publications of the conferences and journals mentioned above from 2016. Using python scripts, we filtered out possible HCML work by looking for keywords. In HCI publications, we searched for all Deep Learning related terms and filtered all publications with at least one match. In AI publications, we searched for human-centered design related keywords. As the final step, we manually went through 320 (including the 50 papers from the search in phase 2) filtered publications and selected 131 papers using the same selection process in phase 2. In total, we analyzed 162 publications in this work.

## 4. Classifying HCML Research

Despite being a nascent field, an attempt to classify HCML related work is beneficial in defining future HCML research boundaries. Categorizing the work can be done along multiple axes, given that HCML consists of many different types of contributions focusing on different domains and different users. While resulting in taxonomy, this also allows us to find desiderata by looking into the missing pieces from the categorization. In this section, we classify the work under several criteria, making it easier for researchers to see the HCML spectrum.

The breadth of our HCML definition also includes work done in areas such as explainable AI (XAI) and data privacy. Sub-research areas such as these have a number of researchers creating different research avenues [11,12,15,17,20,26,28,32,38,39,40,41]. For instance, there are works on developing algorithms and novel DL architectures in XAI to add explainability to the models [42,43,44,45,46]. In comparison, there is also work that considers user experience and user requirements for XAI [7,8,9,10,47], and evaluates algorithms and models with user studies [48]. However, analyzing and categorizing XAI algorithms is not the focus of this paper. Our focus concerns the usability and adoptability of ML systems per the underlying motivation of HCML.

### 4.1. Contribution Varieties

#### 4.1.1. User Studies

Per the HCML definition, it is desirable always to keep the human at the center when developing ML systems. This includes understanding the user requirements, the inputs for the iterative development from a feedback loop, and the evaluation. This can be achieved through many approaches; however, user studies are among the most frequently used approaches to infuse user input into the development. Many existing works in literature have successfully utilized user studies in their development process. Some approaches conduct evaluations and comparisons between existing systems through user studies; however, we will explore work that presented these user studies as the main contribution in this section.

The work of Cai et al. [49] uncovered the requirements of an AI assistant for use in the medical domain. They conducted a thorough study with 21 pathologists to understand how non-AI-experts interact with an AI-tool [49]. They kept the pathologists’ involvement active throughout the AI assistant’s development and presented the findings in a detailed manner. These efforts can be said to make an excellent contribution to HCML. In contrast, to engage the human throughout the process, Liebling et al. [50] conducted a study only to understand the users’ needs for AI language translation applications. Luger et al. [51] conducted a set of interviews to understand people’s perception of voice assistants from tech giants. Since they conducted the study in 2016, it may be outdated. Assistants have evolved significantly over the last four years. In the same area, Candello et al. [52] ran a study to identify the effects of typefaces when communicating with chatbots. In a case study, Yang et al. [53] explored ways to sketch Natural Language Processing (NLP) powered user experiences and presented a wizard-of-oz type NLP rapid prototyping method. Yang et al. [54] studied the affective aspect of conversational agents. In a comparison study, Diaz et al. [55] analyzed age-biases in algorithms related to sentiment analysis. Gero et al. [56] conducted a study to understand mental models people build regarding AI using an AI-game. Schaekermann et al. [57] conducted a study to compare two AI assistants that classify medical time series data.

Apart from studies to understand aspects of specific applications, there have been attempts to understand user concerns, such as explainability and fairness, that later translate into ML models’ features. One comprehensive study [48] has been conducted to explore current practices of explainable-AI in the industry to understand the desirable techniques for real-world usage. Another study [58] attempts to identify gaps between current XAI algorithmic work and practices towards user-centered XAI. In a similar study using industry personnel [59], Holstein et al. compared AI-fairness practices in literature and the real world. In a user study [60] conducted with people from the Amazon Mechanical Turk (https://www.mturk.com/, accessed on 1 April 2021), the trust of an AI model based on stated vs. real-world accuracy of the model is explored. Another user study has been conducted while attempting to find general principles for interpretability [61]. They specifically investigate the interpretability of human-simulatable machine learning models. There have been other studies carried out to explore design approaches for interactive ML tools for non-expert ML engineers to develop models quickly [62]. As opposed to a generic interactive ML tool, Krening et al. [63,64] conducted a user study to investigate specific aspects related to interactive reinforcement learning tools for novice ML engineers. In a completely different attempt to mix a UX team with an AI team, Kayacik et al. [65] present a study on how the teams from two distinct domains interacted to create an AI-Music application. Hong et al. [66] investigated how users conceptualize, experience, and reflect on their engagement in machine teaching.

Beede et al. [67] conducted a real-world study to evaluate a Deep Learning system deployed to detect diabetic retinopathy. A study conducted by Santhanam et al. [68] investigated the effects of cognitive biases when evaluating the outputs of conversational agents. Lin et al. [69] investigated the better approach for collaborative ideation from a physical robot and a virtual agent. Pfau et al. [70] investigated the appropriateness of using bots in games when real players drop due to various reasons. Madaio et al. [71] attempted to understand the role checklists play in AI ethics using 48 practitioners. Xu et al. [72] attempted to investigate children’s perception of conversational agents available in smart devices. Smith-Renner et al. [73] studied how automatically generated explanations of ML models shape users’ perceptions of ML models. Völkel et al. [74] explored how to mislead chatbots in profiling users. A study conducted by Kaur et al. [75] found out that ML practitioners often over-trust and misuse interpretability tools. Alaqaraawi et al. [76] evaluated saliency maps, a popular explanation technique for CNN-based classification algorithms, with a user study. Ishibashi et al. [77] investigated audio visualization techniques for audio related interactive machine learning applications. Das et al. [78] explored whether humans can improve performance using explainable AI capabilities with computer techniques that surpass humans. Drozdal et al. [79] attempted to discover what is vital to ML engineers’ trust in the Auto ML system. Ohn-Bar et al. [80] studied an indoor navigation interface for blind people. Dodge et al. [81] investigated how explanations impact people’s fairness judgment. Yang et al. [82] studied how visual explanations affect end-users’ appropriate trust in ML.

#### 4.1.2. Applications

This section explores the literature that mainly focuses on AI application development and follows HCML principles at one or more points in the development-life-cycle. For example, this could be in the form of a user needs survey, interviews, or evaluating user studies. ‘The Bach Doodle’ is an attempt to develop a web-based music-harmonizing app for large scale deployment [83] where user feedback was used to evaluate the system and ensure reliability before public release. Frid et al. [84] developed an AI background music generation app for video creators where they integrated human input before, during, and after the development. In the same domain, Louie et al. [85] investigated how to adjust an AI music generation tool to minimize user burden with user experiments. The HCML approach has been used in developing assistive technologies. For example, Balasubramanian et al. [2] carried out user experiments to understand the user perspective to develop an assistive AI tool for the blind to grasp non-verbal cues. Kacorri et al. [86] also developed an assistive tool for visually impaired people to get information about visual objects using Deep Learning techniques. Similarly, Feiz et al. [87] used AI technologies to develop an application enabling blind people to write on printed forms. Lee et al. [88] attempts to improve object recognition based applications for blind people by leveraging the hand as a point to consider when focusing on an object in a frame. Zhao et al. [89] developed a face recognition tool for visually impaired people to identify their friends.

Fast et al. [90] attempted to create a camera and fiction-text-input based application to detect human activities. Xu et al. [91] developed and evaluated a new chatbot trained on Twitter conversations to be deployed on social media for customer service. EgoScanning [92] is an application to fast forward first-person recorded videos by being content-aware using object detection techniques. Presenting a unique concept, Kimura et al. [93] developed an application (SottoVoce) to decode speech utterances without voice using ultrasound and Deep Learning. Aila [94] is an application that acts as a document labeling assistant and uses attention-based deep neural networks to perform it. Alghofaili et al. [95] present a Deep Learning based application to pull up navigation aids in Virtual Reality (VR) environments by using gaze patterns. Wu et al. [96] developed an AI-powered tool to assist people with dyslexia to write posts on social media. Guo et al. [97] developed an application to visualize event sequence predictions of multiple records. They used inputs from machine learning practitioners before development and used eighteen participants to evaluate the system. Roffo et al. [98] developed a tool to conduct a standard psychiatric test to assess attachment in children for the first time automatically. VizML [99] is an application made to visualize recommendations using neural networks. Laput et al. [100] developed a system to detect hand activities using sensors available in smartwatches by using a convolutional neural network. SmartEye [101] is an application that assists smartphone users in taking good photos using a view proposal network. McCormack et al. [102] developed an AI application that communicated with improvising musicians in collaborative environments. Swire [103] is an application developed to retrieve user interfaces using sketches. Gamut [104] is a design probe to understand how data scientists understand ML models.

The DeepWriting [105] application used generative neural networks to edit handwritten text, and the paper also published a dataset. A tone-aware chatbot was created by Hu et al. [106] for customer care on social media. Kimura et al. [107] developed a system to augment visual experiences by projecting around the primary display using generative models. Huber et al. [108] developed an image-grounded conversational agent that uses visual sentiment cues. Oh et al. [109] developed an application, DuetDraw, to draw in collaboration with an AI. CheXplain [110] is a tool designed for physicians to understand AI-based analysis on chest X-ray images. Wu et al. [111] developed a Deep Learning system to predict and diagnose user engagement with mobile user interfaces. EarBuddy [112] is an application to control wireless earbuds via interactions on a person’s face. PenSight [113] is a tool where a camera is attached to a tablet pen to create seamless hand inputs using both hands. OralCam [114] is a smartphone app for users to self-examine oral health using Deep Learning. EmoG [115] is a generative tool to support storyboarding by incorporating emotional expressions. Wu et al. [116] developed a system for acoustic activity recognition targeting low user burden by using self-supervised learning techniques. ReCog [117] is a mobile app for blind users to recognize personal objects by letting them train a neural network with their photos. Mirror Ritual [118] is an effective mirror that displays a generated poem to engage the user while conceptualizing an emotional state.

Iconate [119] is a tool created to generate complex icons based on text queries. Sun et al. [120] present a system to aid model selection for demand forecasting. Im et al. [121] attempted to create an application to derive social signals for online social platforms computationally. Xiao et al. [122] attempted to develop a prototype of an active-listening chatbot to interview people. WorldGaze [123] tries to leverage the existing cameras of smartphones to include gaze information to enrich voice assistants. Jensen et al. [124] present a system to generate feedback for teachers in classrooms automatically. ARMath [125] is a Deep Learning powered tool for children to discover mathematical concepts with real-life objects with augmented reality. MaraVis [126] is a Deep Learning powered tool for real-time urban marathon visualization and coordinated intervention. Silva [127] is a tool to find out potential sources of unfairness in datasets or ML models. An NLP-powered tool was developed by Wambsganss et al. [128] for students to develop argumentation quality in writing by providing feedback. Sterman et al. [129] developed a tool to visualize and model writing styles using Deep Learning. Opisthenar [130] is a Deep Learning based tool to recognize head poses and finger tappings. Zhang et al. [131] propose several techniques to correct errors more efficiently when typing on mobile phone keyboards. Bylinskii et al. [132] created a tool to predict the relative importance of elements in graphics and visualizations. Cami et al. [133] introduce a new pen input space using hand postures. Sketchforme [134] is a tool to generate intricate sketches based on text descriptions.

Lip-Interact [135] is a tool to provide silent voice commands via lip movement. CodeMend [136] is a tool to support search and integration of code for programmers. HairBrush [137] is an interactive 3D hair modeling system. LabelAR [138] is an augmented-reality-based tool to label objects for computer vision in a novel way. ViZig [139] is an application developed with semi-supervised training to find anchor points in instructional videos. AlterEgo [140] is a wearable silent speech interface that allows users to converse silently. Alcove [141] is an assistive comic reading tool for people with low vision. iSeqL [142] is a tool designed for the rapid construction of customized text mining models. Creative Sketching Partner [143] is a proof-of-concept intelligent interface to inspire designers while sketching. Scones [144] is a tool that generates and modifies sketches iteratively according to text descriptions. CQAVis [145] is an application to filter high-quality comments from online community question answering forums. Zhelezniakov et al. [146] investigated the requirements for pen-centered mathematical expression recognition applications. SaIL [147] is an assistive web navigation tool that injects important ARIA landmarks automatically. Grover et al. [148] designed and evaluated intelligent agents to improve productivity and focus at work. Cartograph [149] is a visualization system that uses knowledge from Wikipedia to create thematic maps. Kulahcioglu et al. [150] created an affect-aware color palette selection tool for word cloud generation. VASTA [151] is a vision and language-assisted programming-by-demonstration system for smartphone task automation. Davis et al. [152] is an intelligent drawing partner that can improvise and collaborate on abstract sketches. Piano Genie [153] is an intelligent controller for non-musicians to improvise on the piano. SViM [154] is an adaptive screen-magnifier tool for people with low vision to watch videos. Lee et al. [155] developed a tool to assess the quality of rehabilitation exercises. Sun et al. [156] created a tool for developers to learn about the dataset while labeling. These AI applications have incorporated the human aspect in ML systems’ development and can thus be classified as HCML research.

#### 4.1.3. Algorithms

Some work [157,158] has been done to improve the interpretability of the Deep Learning algorithms for general and computer vision purposes by employing user evaluations to assess intended outcomes. Arendit et al. [159] developed an efficient image labeling tool with user evaluations to support its intended performance. In a similar tool to efficiently label audio samples, Kim et al. [160] also follow a similar human-centered approach to evaluate success. Although these can be identified as tools, the key contribution, as described in the publications, is developing and evaluating the algorithm. We classify these papers as algorithmic contributions that follow HCML principles. Banovic et al. [161] developed a new weakly supervised algorithm based on inverse reinforcement learning to detect and generate human behaviors. Fridman et al. [162] developed an algorithm to predict the driver’s states using videos of driver glances. Crowdverge [163] is an algorithm to figure out whether crowds would agree on one answer for visual question answering tasks. Huang et al. [164] developed an algorithm to augment static panorama images through a realistic audio assignment. Guzdial et al. [165] developed an algorithm to design levels in games like Super Mario and investigated the user aspect with level designers. Kim et al. [166] present a Deep Learning based algorithm to estimate eye-gaze with low latency. Seemo [167] is a framework developed to map emotions into vector representations using representation learning techniques.

Ryolai et al. [168] explored ways to assign laughter to tangible objects. Yuan et al. [169] trained a deep learning model to predict the scannability of webpage content. Cogam [170] tried to generate explanations for machine learning models by incorporating the desired cognitive load. Lai et al. [171] created a technique to annotate visualizations according to text descriptions automatically. Soundr [172] used Deep Learning to figure out the user’s spatial location and head orientation using voice. Duan et al. [173] developed a technique to optimize UI interfaces automatically with error correction. Pfau et al. [174] looked at how Deep Learning techniques can improve dynamic difficulty adjustments in games. Bassen et al. [175] developed a reinforcement learning algorithm to optimize educational activities in online courses. Donkers et al. [176] developed a recommendation and explanation method and evaluated the quality with user studies. Arent et al. [177] present a technique that generalizes parallel coordinated visualization techniques to sequences of learned representations. Eshan et al. [178] created a technique to generate rationales automatically. Le et al. [179] developed a model to identify fingers in capacitive touchscreens. CoSummary [180] is a technique to fast-forward surgical videos adaptively. Micallef et al. [181] created a technique that uses interactive visualization to elicit the need of domain experts to improve the accuracy of prediction models. Athukorala et al. [182] created a novel adaptation technique for search engines. Mittal et al. [183] proposed an architecture to generate emoticons using multi-modal input. Weber et al. [184] present a combination of manual and automated Deep-Image-Prior-based image restoration techniques. Schlegel et al. [185] illustrate a model-agnostic visual debugging workflow for multi-target time series classifications.

#### 4.1.4. Non-AI-Expert Tools

Apart from applications that use AI in the back-end where the users are not exposed to the underlying technology or AI model, some tools allow users to interface with the AI algorithms [186,187,188,189,190,191]. These tools are often referred to as non-AI-expert tools or non-expert tools in general. For instance, a voice assistant is an AI application for the user to engage verbally. The user might not even know that AI algorithms run in the back-end. However, a customized tool designed for a physician to interact with an ML model is a non-AI-expert tool where the non-AI-expert manipulates AI algorithms without having AI-specific knowledge. A non-AI-expert’s level of expertise can range from none, such as a lawyer with no AI background, to a moderate level, such as a novice ML engineer. These variations in knowledge require designers to determine the correct level of abstraction for specific user groups. Here, we look at several attempts to develop such non-AI-expert tools following the HCML approach. SMILY [192,193] is a prime example of a non-AI-expert tool. It allows pathologists to change parameters of an ML model to search medical images. The What-If Tool [194] is designed for non-expert ML engineers to carry out interactive probing across different input variations. They have conducted an evaluation study with ML students to validate their tool. To make machine learning models accessible to non-AI-experts, Ramos et al. [195] showed how to leverage intrinsic human capabilities of teaching to teach machines to do machine learning.

#### 4.1.5. Principles and Guidelines

Among the HCML literature, this category pertains to research that compiles design guidelines and principles for HCML or provides assistance to build HCML products and services. These works stem from different intentions, such as guidelines for developing intelligent user interfaces, visualization, prototyping, and human concerns in general. Amershi et al. [196] present a set of guidelines resulting from a comprehensive study conducted with many industry practitioners that worked on 20 popular AI products. Some approaches have been focused on deriving requirements and guidelines for planned sandbox visualization tools [197]. One article highlights guidelines related to three areas of HCML, ethically aligned design, tech that reflect human intelligence, and human factors design [198]. Browne et al. [199] proposed a wizard of oz approach to bridge designers with engineers to build a human-considerate machine learning system targeting explainability, usability, and understandability. Some papers attempt to identify what HCML is [200], and discuss how AI systems should understand the human and vice versa. Apart from general perspectives, Chancellor et al. [201] attempted to analyze literature in the mental health-AI domain to understand which humans are focused on such work and compile guidelines to maintain humans as a priority. In a slightly different layout, Ehsan et al. [202] attempted to uncover how to classify human-centered explainable AI in terms of prioritizing the human. Wang et al. [203] also tried to design theory driven by a user-centered explainable AI framework and evaluate a tool developed with actual clinicians. Schlesinger et al. [204] explored ways to build chatbots that can handle ‘race-talk’. Long et al. [205] attempts to define learner-centered AI and figure out design considerations. Yang et al. [206] explore insights for designers and researchers to address challenges in human–AI interaction.

#### 4.1.6. Survey Results

**Figure 5:**
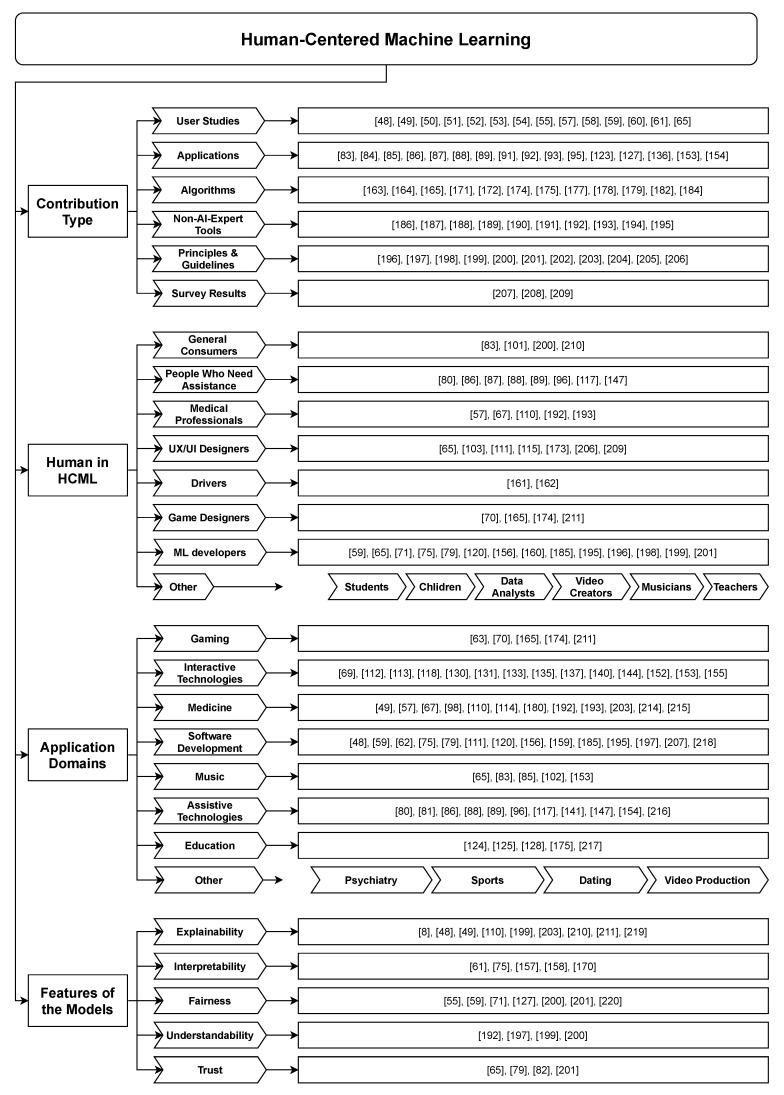
Classification chart. Each category has a few representative examples.

Some work has focused on presenting analyzed results of surveys conducted towards different goals Figure 5, such as understanding human perspectives before developing solutions. For example, Cai et al. [207] present many hurdles software engineers faced working in the machine learning field through a thorough survey and analysis. Other survey studies [208] focused on finding out people’s opinions about delegating tasks to AI agents to help ML engineers and product developers. Dove et al. [209] surveyed to understand how design innovation is practiced in the ML domain in terms of user experience.

### 4.2. The ‘Human’ in HCML

The main component of HCML is the Human and thus elevating the significance of the human. The ‘Human’ in HCML is defined across varying ML expertise levels, ranging from no ML background to an expert ML scientist. The Human in HCML can also be involved in various stages of the ML system development process in different capacities. For instance, the focus may be on the end-user, the developer, or the investor. One could focus on a certain user-aspect when developing a product or service [83,160]; another could be determining design principles for a particular ML system optimizing usability and adoptability [48,65,196]. The multidimensionality of what is considered Human within HCML contributes to the complexities within the field.

Considering the works that focused on the user side, some researchers catered to general end-users or consumers [83,101,200,210], while others on specific end-users. Examples for these include people who need assistance [2,80,86,87,88,89,96,117,147], medical professionals [57,67,110,192,193], international travelers [50], Amazon Mechanical Turk [60,99], drivers [161,162], musicians [102], teachers [124], students [128], children [72,125], UX designers [65,115,206,209], UI designers [103,111,173], data analysts [97], video creators [84], and game designers [70,165,174,211]. Apart from focusing on a specific user group, some have tried to understand multiple user-perspectives from ML engineers to the end-user [48]. Some of the prior works that target the developer as the human focus on novice ML engineers to help them develop ML systems faster [62,197]. Notably, the majority of works that target the developer side focused on ML engineers [59,65,71,75,79,120,156,160,185,195,196,198,199,201,207].

### 4.3. Application Domains

Machine Learning works well in many scenarios provided that a relationship exists between the task at hand and the availability of data. This power of making decisions or predictions based on data has empowered ML to infiltrate many other domains, such as medicine, pharmacy, law, business, finance, art, agriculture, photography, sports, education, media, military, and politics. Given that the majority in those sectors are not AI experts developing AI systems for them requires us to investigate the human aspect of such systems. Our analysis shows that application domains have specifically targeted gaming [63,70,165,174,211], interactive technologies [69,112,113,118,130,131,133,134,135,137,140,144,152,153,155,212,213], medicine [49,57,67,110,114,180,192,193,203,214,215], psychiatry [98], music [65,83,85,102,153], sports [126], dating [60], video production [84], assistive technologies [2,80,86,88,89,96,117,141,147,154,216], education [124,125,128,175,217], and mainly software and ML engineering [48,59,62,75,79,111,120,156,159,185,195,197,207,218] based on our selected literature.

### 4.4. Features of the Models

Features of AI models addressing the concerns of users to improve the usability and adoptability of AI systems such as explainability, interpretability, privacy, and fairness have been the focus of many HCML related work [6,12,20,26,58,73,76,81,104,178,219]. This is not surprising, given the history of XAI research area dates back to 1980s [220,221]. In a comprehensive study, Bhatt et al. [48] investigated how explainability is practiced in real-world industrial AI products and presents how to focus explainability research on the end-user. Focusing on game designers, Zhu et al. [211] discuss how explainable AI should work for designers. A study [210] used 1150 online drawing platform users and compared two explanation approaches to figure out which approach is better. Ashktorab et al. [222] explored explanations of machine learning algorithms concerning chatbots. Although explainability is not the main focus, some research [49,199] investigated the explainability aspect when developing ML systems. Another work [202] tried to investigate who is the human in the center of human-centered explainable AI. In addition, there exists work that tried to bring a user-centered approach to XAI research [8,203]. Chexplain [110] worked on providing an explainable analysis of chest X-rays to physicians. Das et al. [78] attempted to improve humans’ performance by leveraging XAI techniques.

While explainability tries to untangle what is happening inside the Deep Learning black boxes, interpretability investigates how to make AI systems predictable. For instance, if a certain neural network classifies an MRI image as cancer, figuring out how the network makes such a decision falls into explainability research. However, an attempt to build a predictable MRI classification network where a change of network’s parameters results in an expected outcome falls into interpretability research. There have been attempts [157,158,170] to develop novel interpretability algorithms using human studies to validate if those algorithms achieved the expected results. Isaac et al. [61] studied what matters to the interpretability of an ML system using a human study. Another study [75] figured out that ML practitioners often over-trust or misuse interpretability tools.

Apart from these two common DL features, some other work considered the aspects of fairness [55,59,71,127,200,201,223], understandability [192,197,199,200], and trust [60,79,82,201]. Fairness represents the degree of bias in decisions, such as gender and ethnic skews, that influence the predictive model. For instance, gender and ethnic biases in the models can cause serious impacts on certain tasks. Understandability is a slightly different feature from explainability. While explainability shows how a model makes a certain decision, understandability tries to show how a neural network works to achieve a task. Trust refers to a subjective concern where the user’s trust towards the decisions made by a certain model is studied.

## 5. Discussion

**Figure 6:**
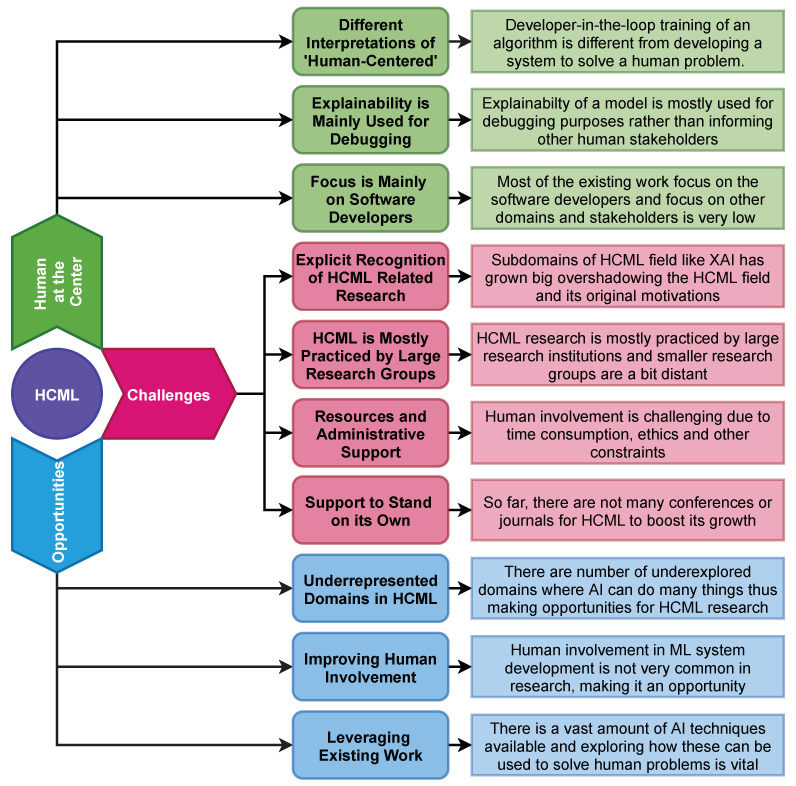
Summary of the Discussion Section. This figure represents the points discussed, and each point is followed by the main idea behind it.

The breadth and depth of the classification presented in the previous section show that HCML grows in many branches despite being a relatively new field of research. However, given the variations in the field, the young age, and the rapid growth of AI, the HCML field has numerous research gaps, challenges, and confusions that are interesting to be discussed and analyzed. For example, a standard definition has yet to be agreed upon, resulting in some liberal interpretations per researchers’ prerogative and the level of abstraction. Here we provide a discussion (see Figure 6 for a summary of this section) concerning these gaps, challenges, and confusions under several subtopics. Apart from this, it is also important to discuss the scope and limitations of current research to identify the opportunities for future work.

### 5.1. Human at the Center

#### 5.1.1. Interpretation of ‘Human-Centered’

One of the main confusions we uncovered within HCML literature is identifying who the ‘human’ is in HCML and how the ‘human’ is involved. As described in Section 4, ‘human’ could be any stakeholder of the work, ranging from the owner of the research institute to the end-user. However, merely any kind of human involvement will not make a particular AI research an HCML approach. The HCML approach depends on the phase in which humans interact with the development life cycle, the purpose of the interaction, and how they interact. For example, while many agree that HCML exists to create usable and adaptable systems for users, others [224,225] argue that an ML engineer interfering with the training loop when adjusting parameters is recognizable as HCML. The latter perspective may be interpreted as a valid HCML approach assuming that interferences from ML engineers contribute to improvements in usability and adoptability, apart from accelerating and optimizing the training process. However, creating a tool centered on ML engineers’ needs to accelerate the training process differs from claiming HCML is about the human interfering with the training process.

#### 5.1.2. Explainability Moving from Non-Experts to Experts

A thorough study conducted with leading industry research teams stated that, despite being created to cater to user needs, ML engineers mainly use explainability research for debugging purposes in practice [48]. The study investigates how to move the explainability research to inform end-users rather than focus on implementation improvements for engineers. These practical issues may already exist in other HCML research areas or emerge soon if not addressed clearly.

#### 5.1.3. Too Much Focus on Software Developers

From automated data labeling tools to interactive machine learning tools, many HCML related work made ML engineers the ‘human’ at the center. The majority of the work we analyzed targets software engineers working in the ML domain. The remainder focuses on an array of different stakeholders and makes software engineers the most focused ‘human’ in HCML research. These findings also further validate the results of Bhatt et al. [48]. Therefore, it is desirable to expand research to other non-AI-experts.

### 5.2. Challenges to HCML

After analyzing the confusions, problems, and gaps in HCML research, we scaffolded the underlying challenges that must be addressed in future HCML guided research. These challenges hinder the rapid and coherent development of HCML research. We discuss these challenges under the four categories below.

#### 5.2.1. Explicitly Recognizing as HCML Research

In our search for literature and forming the definition, we understood the confusions that occur when labeling work as HCML. For instance, although XAI (explainable AI) serves the overall goals of HCML, it is an independent field and is not mainly categorized as HCML work. In XAI, research is being conducted to add explainability to models; however, most publications do not follow a human-centered approach. This has reduced the number of works in studying the end-user or human aspect of explainable AI algorithms. However, there are works that attempt to bring an HCML approach into XAI [8,47,58]. Although XAI is the best example, this problem exists in research on other user-concerns, such as interpretability, privacy, and fairness. We identified that one major reason for this is the lack of extension of the HCML practices to small research teams.

#### 5.2.2. Extending HCML to Small Research Groups

The problem of missing end-user involvement in HCML work seems to be in research interests. Machine Learning researchers are focusing mainly on algorithm development and optimization while the human-centered approach is practiced mainly by Human–Computer Interaction (HCI) researchers. Therefore, collaborations between HCI and ML research produce good HCML outputs. These collaborations and facilitation seem well-established within large industry and academic research groups, such as Google Research (https://research.google/, accessed on 1 April 2021), Microsoft Research (https://www.microsoft.com/en-us/research/, accessed on 1 April 2021), MIT (https://www.mit.edu/, accessed on 1 April 2021), and Stanford (https://www.stanford.edu/, accessed on 1 April 2021). Therefore, their efforts have to be appreciated. However, this facilitation and collaborations are not well-established within small research groups. As a result, it is not common to see small research groups publish good HCML work. We identified that extending this HCML vision to smaller research groups will accelerate performant outputs with the necessary usability and adoptability.

#### 5.2.3. Resources and Administrative Support

The HCML research we analyzed had several possible improvements that could be observed in terms of the completeness of work. However, achieving this will entail challenges. For instance, some research uses human inputs to identify user requirements, whereas other research used human users for evaluations. Apart from a small selection of works, most of the corpora’s human-centered practice is limited to a particular stage of the system development life cycle. While understandable that it is desirable to keep the human at the center throughout the entire development or design cycle, there are many practical hurdles. Access to a group of specific people, institutional ethics approvals, time consumption, meeting internal and publication deadlines are some hurdles to overcome to maximize human involvement throughout the process. These hurdles may result in a lack of human involvement or motivate conducting a user study for superficial purposes only. These reduce the quality of outcomes of HCML research, hence decelerating the development of the field.

#### 5.2.4. Support to Stand on Its Own

Many established research fields have their own high rated conferences and journals with high impact. For example, AI and ML have many conferences including NeurIPS (https://nips.cc/, accessed on 1 April 2021) and AAAI (https://www.aaai.org/, accessed on 1 April 2021), and HCI has its own conferences, such as CHI (https://dl.acm.org/conference/chi, accessed on 1 April 2021) and IMWUT (https://dl.acm.org/journal/imwut, accessed on 1 April 2021). However, HCML does not have a popular conference or a journal yet. Although venues like IUI (https://dl.acm.org/conference/iui, accessed on 1 April 2021) and TiiS (https://dl.acm.org/journal/tiis, accessed on 1 April 2021) include HCML work in their broader scope, those venues are not solely focused on the HCML field. Having dedicated publication venues (examples include ASSETS (https://dl.acm.org/conference/assets, accessed on 1 April 2021), AHs (https://augmented-humans.org/, accessed on 1 April 2021) for a field can help streamline the research, as well as assist and solve many challenges mentioned in this section. We believe that HCML has a certain maturity to be represented in its own publication venue, despite being an emerging field.

### 5.3. Opportunities for HCML and the Future

#### 5.3.1. Underrepresented Domains in HCML

At the inception of HCML, the main focus was on the end-user as in Human-Centered Design. While the ‘human’ now represents a broader definition, there has been a definite shift in focus to the software or ML engineers. Therefore, it is desirable to see more research targeted to end-users in other general or specific domains. In analyzing industry-specific AI trends and the adoption of HCML driven research, there are apparent shortcomings. AI usage in agriculture is a real need [226]; however, publications related to HCML and agriculture were scarce. Similarly, in medicine, the HCML aspect of medical AI applications is rare, as algorithmic development and optimization dominate the field. Given that medicine is a field filled with non-AI-experts working with critical situations, a human-centered approach will ensure the novel algorithms can be packaged as applications that are useful and adaptable, as Cai et al. [192] demonstrated. Other domains such as law, music, art, chemistry, education, finance, media, politics, photography, and the movie industry face the same under-representation problem in HCML research.

#### 5.3.2. Improving Human Involvement

Exciting work with AI has been produced in law, such as using ML to help judges decide bail [227]. However, such work did not fall within our HCML definition scope due to the lack of investigation on the usability and adoptability of their application. They have either hypothesized the user needs based on assumed user behavior or failed to present how they derived the end-user requirements, namely the judges. This problem of researchers presuming end-user requirements without purposeful investigation is prevalent in many other works [228,229]. We believe that this practice does not work efficiently toward developing usable and adaptable AI systems. Therefore, it is imperative that researchers involve humans in the development process.

#### 5.3.3. Leveraging Existing Work

AI technologies evolve fast and generate usable models and tools in the real-world. Many of these significant works have not yet been studied in terms of real user aspects. An opportunity exists to quickly leverage existing AI technologies to design, develop, and validate real-world AI systems successfully. For instance, it could be investigating models or even useful tools created by other researchers [187,230].

Going beyond small to medium scale, current trends, and innovations in the broader field of high-performance AI suggest a rising significance of HCML. For instance, Deep Learning models are computed at the current technological limitations of available hardware. Models such as GPT-2 [231] have 1.5 billion parameters that give incredibly good results on Natural Language Processing (NLP) tasks. However, it has recently been outperformed by GPT-3 [232], a 175 billion parameter network, which is the largest Deep Learning model to date. These large networks demonstrate that having more parameters improves the model performance and detracts interest from innovation and optimization techniques driven improvements. Few institutions, let alone researchers, have access to the hardware resources to train such monolithic networks. This growing reliance on raw computational power elevates the need to investigate the real-world application aspect of existing models over hardware-dependent improvement in benchmarked accuracy. The arrival of multi-billion parameter networks also brings vast opportunities to HCML, given the rotation of interest from pure performance towards the human aspect of modern AI.

## 6. Conclusions

Human-Centered Machine Learning (HCML) is an emerging field of research parallel to the exponential growth of Deep Learning (DL) research for several reasons. Some of the reasons are the frequent application of Artificial Intelligence (AI) models in the real world, an increasing number of interactions between humans and AI, professionals raising concerns regarding the black-box nature of DL, and questions about the AI models’ reliability in critical application scenarios. Even though HCML is a nascent field, its origin dates back decades, and the exact term itself is more than a decade old. However, with the AI boom in the early 2010s and the adaptation of Deep Learning techniques, HCML re-emerged with different and modern concerns. Those are mostly usability and adoptability related concerns such as user experience, explainability, understandability, interpretability, privacy, security, fairness, and reliability.

Many research groups started working around HCML with different interpretations of the term. Hence, in this work, we present a working definition for HCML derived from analyzing those interpretations. We followed a systematic literature review approach to make the literature search repeatable and expand its search base. We classified 162 HCML publications we selected in terms of contribution type, application domain, the ‘human’ at the center, and the models’ features. Our search and the analysis of prior work enabled us to understand the spectrum of HCML while identifying the confusions, research gaps, and possible research directions. In conclusion, HCML is an emerging but fast-growing field, given the foundation field—AI—is snowballing. HCML has sub research fields that stand independently, hence reducing the focus on core concepts of HCML. Given the early stage of the field, there are confusions, problems, and opportunities for development, which we discussed in this work. Deep Learning is approaching the limitation of current hardware’s abilities while user-aspect investigations are lagging. The field of HCML can bridge the critical divide between model performance and end-user adoption. We believe that this is the prime time to research HCML, given the tremendous opportunities.
